# An R package for analyzing and modeling ranking data

**DOI:** 10.1186/1471-2288-13-65

**Published:** 2013-05-14

**Authors:** Paul H Lee, Philip LH Yu

**Affiliations:** 1 School of Public Health/Department of Community Medicine, The University of Hong Kong, Room 624-627, Core F, Cyberport 3, 100 Cyberport Road, Hong Kong, Hong Kong; 2 Department of Statistics and Actuarial Science, The University of Hong Kong, Hong Kong, Hong Kong

**Keywords:** Distance-based model, Luce model, Multidimensional preference analysis, Visualization, Weighted distance

## Abstract

**Background:**

In medical informatics, psychology, market research and many other fields, researchers often need to analyze and model ranking data. However, there is no statistical software that provides tools for the comprehensive analysis of ranking data. Here, we present pmr, an R package for analyzing and modeling ranking data with a bundle of tools. The pmr package enables descriptive statistics (mean rank, pairwise frequencies, and marginal matrix), Analytic Hierarchy Process models (with Saaty’s and Koczkodaj’s inconsistencies), probability models (Luce model, distance-based model, and rank-ordered logit model), and the visualization of ranking data with multidimensional preference analysis.

**Results:**

Examples of the use of package pmr are given using a real ranking dataset from medical informatics, in which 566 Hong Kong physicians ranked the top five incentives (1: competitive pressures; 2: increased savings; 3: government regulation; 4: improved efficiency; 5: improved quality care; 6: patient demand; 7: financial incentives) to the computerization of clinical practice. The mean rank showed that item 4 is the most preferred item and item 3 is the least preferred item, and significance difference was found between physicians’ preferences with respect to their monthly income. A multidimensional preference analysis identified two dimensions that explain 42% of the total variance. The first can be interpreted as the overall preference of the seven items (labeled as “internal/external”), and the second dimension can be interpreted as their overall variance of (labeled as “push/pull factors”). Various statistical models were fitted, and the best were found to be weighted distance-based models with Spearman’s footrule distance.

**Conclusions:**

In this paper, we presented the R package pmr, the first package for analyzing and modeling ranking data. The package provides insight to users through descriptive statistics of ranking data. Users can also visualize ranking data by applying a thought multidimensional preference analysis. Various probability models for ranking data are also included, allowing users to choose that which is most suitable to their specific situations.

## Background

Ranking data arises when a number of items are to be ranked. By the nature of the ranking data, they can be divided into two types. The first type is characterized by a small number of items to be ranked, and they frequently represent the preference of these items among a group of judges (individuals). These items can be candidates in an election [1], one’s place of living [2], choice of occupations [3,4], medical treatment [5], and so on. In analyzing these data, the focus is on the judges’ perception and preference of some specific (or all) items. In recent years, this type of ranking data have also becoming more popular in the medical literature, particularly in health economics [6-10] and medical informatics [11].

The second type of ranking data is characterized by a large number of items, and they frequently represent the ordering of these items in which researchers would like to determine or predict which items were ranked at the top positions. Examples of such ranking data include search engine results [12], integration of microRNA and mRNA [13], and consumer behavior in e-commerce applications [14]. Due to the large number of items, these ranking datasets often contain missing or tie rankings, which are impossible to analyze without computers. With the decreasing cost of powerful computers, more researchers have paid attention to this type of ranking data, especially those in machine learning and knowledge discovery.

Analyzing and modeling ranking data is an efficient way to understand people’s perceptions and preferences for different items. Over the years, besides statistical tests for hypothesis testing [15], various models have been developed for ranking data, including the Luce model [16], distance-based model [1], ϕ-component model [17] and weighted distance-based model [18,19].

The maximum likelihood estimator (MLE) of the aforementioned models does not have a closed form, yet the MLE can be obtained using iterative algorithms. However, at present, only summary statistics and a visualization of ranking data are available (partially and indirectly) in some statistical software (for example, procedure MDPREF in SAS), not to mention hypothesis testing and probability models for ranking data. The lack of software and tools for analyzing ranking data is not a problem for statisticians who are used to writing programs for their own means. However many scientists are not familiar with programming. We believe that a single package for the analysis of ranking data could offer users a more complete analysis, allowing them to use a single program instead of shifting their ranking datasets from one application to another.

**Figure 1:**
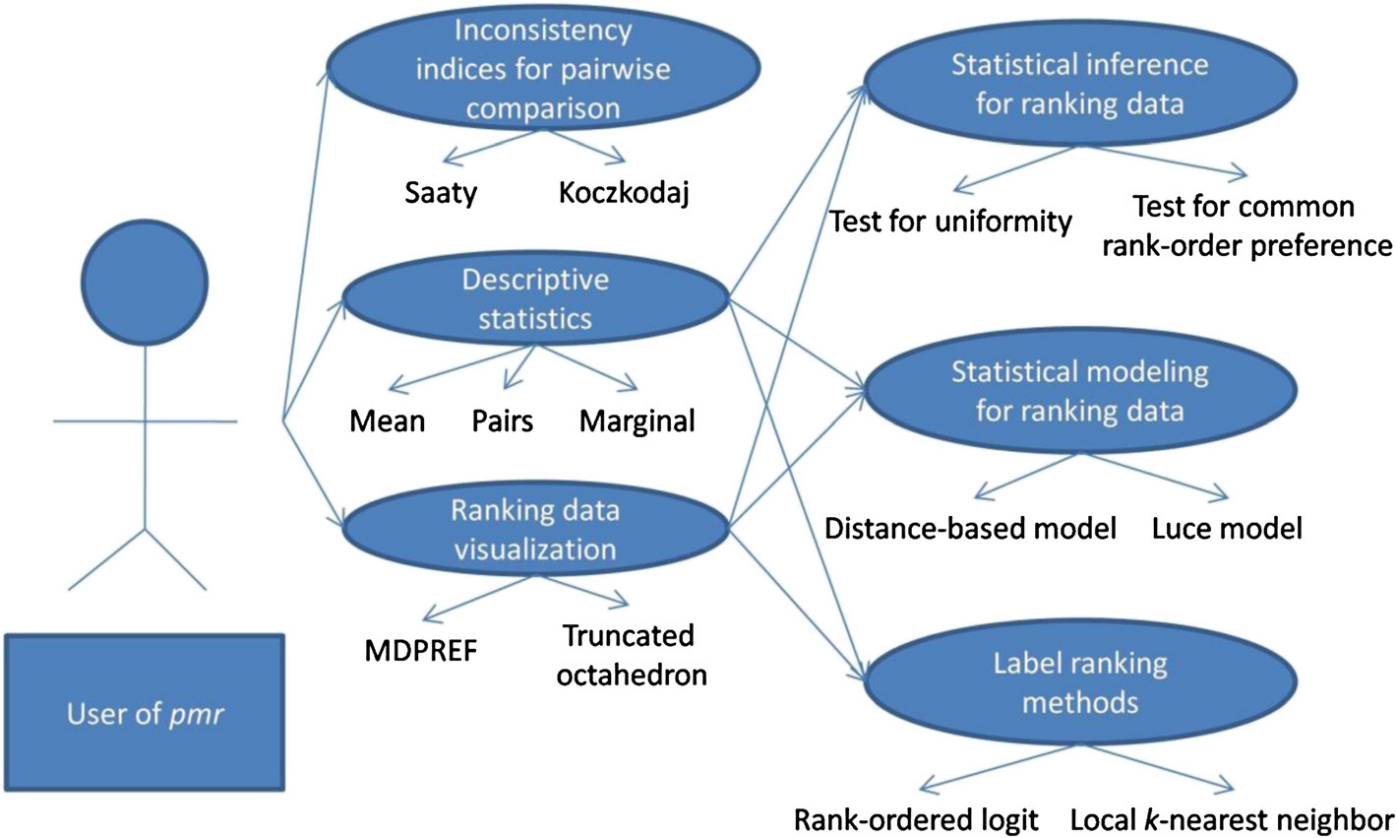
**Use Case Diagram of the *pmr *package.**

R [20], an open-source program for statistical analysis, is gaining in popularity because of its high flexibility. Indeed, users are free to write/use packages for specific purposes. Although many packages are highly relevant to medicine [21,22], there are only a limited number of packages for analyzing and modeling ranking data. There are some basic tools for ranking data, for example the *Kendall* package and the *pspearman* package for the computation of Kendall and Spearman rank correlation. Nonetheless, to the best of the authors’ knowledge, the only statistical model currently available in R is the *RMallow* package (http://cran.r-project.org/web/packages/RMallow) for fitting a mixture of Mallows’ models [23]. Here, we present *pmr* (*p*robability *m*odels for *r*anking data), an R package for analyzing and modeling ranking data with a bundle of statistical tools. A review of statistical analysis for ranking data is given, prior to demonstrating the implementation of *pmr*. The current version of *pmr* and the user manual can be found in Additional files 1 and 2 respectively. In addition, four ranking models are reviewed, namely the Luce model, distance-based model, ϕ-component model, and weighted distance-based model. For more details, readers can refer to [15,24,25]. The use cases diagram of the *pmr* package is shown in Figure 1.

## Implementation

In this section, we give a review of statistical analyses for ranking data. For a better description of ranking data, some notations must be defined. For a set of *k* items, labelled 1,…, *k*, a ranking π is a mapping function from 1,…, *k* to 1,…, *k*, where *π*(*i*) is the rank given to item *i*. For example, *π*(2) = 3 means that item 2 has a rank of 3. The inverse of the ranking function (sometimes referred to as ordering) *π*^-1^(*i*) is defined as the item that has rank *i*. For example, *π*^-1^(5) = 6 means that the item with rank 5 is item 6.

### Descriptive statistics for ranking data

Descriptive statistics give an overall picture of the ranking dataset. Not only do descriptive statistics provide a summary of the ranking dataset, but they also lead us in an appropriate direction to analyze the dataset. Therefore, it is suggested that researchers consider descriptive statistics prior to any sophisticated analysis of ranking data.

We begin with a single measure of the popularity of an item. It is natural to use the mean rank attributed to an item to represent the central tendency of the ranks. Mean rank *m* is defined as the *k*-dimensional vector in which the *j*th entry equals

$$m_{j}=\sum_{i=1}^{k!}N_{i}\pi_{i}\left(j\right),$$

where *π*_*i*_, *i* = 1, 2, …, *k*! represents all possible rankings of the *k* items, *N*_*i*_ is the observed frequency of ranking *i*, and *π*_*i*_(*j*) is the rank given to item *j* in ranking *i*.

Apart from the mean ranks, the pairwise frequencies, that is, the frequency with which item *i* is ranked higher than item *j* for every possible $C_{2}^{k}$ item pairs (*i*, *j*), are also often used. These pairwise frequencies can be summarized in a *k*×*k* matrix called a pair matrix (*P*) in which the (*s*,*t*)^th^ entry equals

$$P_{\mathrm{st}}=\sum_{i=1}^{k!}N_{i}I\left[\pi_{i}\left(s\right)>\pi_{i}\left(t\right)\right],$$

where *I*[·] is the indicator function. Note that *P*_*st*_/*N* represents the empirical probability that item *s* is ranked higher than item *t*. In addition to mean ranks and pairwise frequencies, one can look more deeply into a ranking dataset by studying the so-called “marginal” distribution of the items. A marginal matrix, specifically for this use, is the *k*×*k* matrix *M* in which the (*s*,*t*)^th^ entry equals

$$M_{\mathrm{st}}=\sum_{i=1}^{k!}N_{i}I\left[\pi_{i}\left(s\right)=t\right].$$

Note that *M*_*st*_ is the frequency of item *s* being ranked *t*^*th*^. It is called a marginal matrix because “the *i*^*th*^ row gives the observed marginal distribution of the ranks assigned to item *i*, and the *j*^*th*^ column gives the marginal distribution of objects given the rank *j*.” ([15], page 18).

### Inconsistency indices for pairwise comparisons

According to the Analytic Hierarchy Process [26], a group of judges combine the rankings from different criteria to form a final ranking. The Analytic Hierachy Process has been used to determine the weights of these criteria. First, a pairwise comparison matrix *A*, in which the (*s*,*t*)^th^ entry *a*_*st*_ equals the number of times criterion *s* is preferred over criterion *t*, is computed. The weights are then found as the eigenvalues of the matrix *A*. The reliability of these weights depends on the consistency of the ranking process, which is defined as *a*_*st*_*a*_*tu*_ = *a*_*su*_ for *s*, *t*, *u* = (1,…, *k*). Therefore, evaluating the consistency of the ranking data using A is a crucial task in analyzing ranking data and hence a number of measures have been developed for this purpose. One popular measure is Saaty’s index, which is given by

$$\frac{\frac{\lambda_{\max}-k}{k-1}}{RI_{k}},$$

where λ_*max*_ is the largest eigenvalue of *A*, and *RI*_*k*_ is the average value of $\frac{\lambda_{\max}-k}{k-1}$ for a *k*×*k* random matrix. Another popular measure is Koczkodaj’s index, which equals $\max\left\{\min\left(\left|1-\frac{b}{\mathrm{ac}}\right|,\left|1-\frac{\mathrm{ac}}{b}\right|\right)\right)$ for each triad (*a*, *b*, *c*) in *A*}.

Other consistency indices exist besides these two [27].

### Visualizing ranking data: multidimensional preference analysis

Because ranking data often have a high dimension, visualization is a good first step towards their analysis. Multidimensional preference analysis [28] is a dimension reduction technique that aims to display ranking data in a low-dimensional (preferably 2D or 3D) space. It is applicable to ranking data with five or more items where the dataset cannot be displayed in a 2D/3D plot. Let *X* be an *N*×*k* matrix of ranking data such that *x*_*ij*_ represents the rank of item *j* assigned by judge *i*, centered by the overall mean rank, i.e., (*k* + 1)/2. Suppose the singular value decomposition of *X* is *X* = *UDV*’. A 2D representation of the multidimensional preference analysis denotes the items and judges by the first two columns of $\sqrt{N-1}U$ and $\frac{\mathrm{DV}\prime }{\sqrt{N-1}}$, respectively. Items are usually plotted as points, whereas judges are plotted as vectors from the origin. To give a better graphical display, the length of the ranking vectors can be scaled to fit the position of the items. It is not difficult to see that the perpendicular projection of all *k* item points onto a judge vector will closely approximate the ranking of the *k* items by that judge if the 2D solution fits the data well. Otherwise, we may look for a higher-dimension solution.

### Statistical inferences for ranking data

Apart from exploring ranking data using descriptive statistics and graphs to identify the structure of the data, statistical inferences can be made to test the significance of a data structure. The two most commonly used inferences are the test for uniformity in a set of ranking data and the test for common rank-order preference for two sets of ranking data.

When we say that a ranking dataset is uniform, we mean that all possible rankings have the same probability of being observed. Hence, under uniformity, the expected frequencies of every ranking should be *N*/*k*!, and the standard *χ*^2^ test can be applied to test the uniformity. However, when *k*! is too large compare with *N*, this is not always applicable, because we may encounter rankings with fewer than five observation. In such a case, mean rank, pairs, or marginals can be used to test the uniformity instead of ranking proportions [15]. Note that under uniformity, the expected values of mean rank, pairs, and marginals are (*k* + 1)/2, 0.5 *N*, and *N*/*k* respectively.

Under uniformity, the test statistic when using mean rank, pairs, and marginals are ([15], page 58, Table 3.1)

$$\frac{12N}{k\left(k+1\right)}\sum_{j=1}^{k}{\left(m_{j}-\frac{k+1}{2}\right)}^{2},$$

$12N\left[{\left(\sum_{s>t}^{k}P_{\mathrm{st}}-0.5\right)}^{2}-\frac{{\left(\sum_{s>t}^{k}m_{j}-\frac{k+1}{2}\right)}^{2}}{k+1}\right],$ and

$$N\left(K+1\right)\sum_{s>t}^{k}{\left(M_{\mathrm{st}}-\frac{1}{k}\right)}^{2},$$

and they follow a *χ*^2^ distribution with *k*-1, $C_{2}^{k}$, and (*k*-1)^2^ degrees of freedom, respectively.

The *χ*^2^ test could be used to test for any difference between two ranking datasets. Before doing so, we align the matrix (in the case of pairs and marginals) into a *q* × 1 vector, for both datasets. We can now use the standard *χ*^2^ test. For comparison between three or more ranking datasets, MANOVA-like tests can be used [15].

### Statistical models for ranking data: the Luce model

After conducting a descriptive analysis for ranking data, we may have some understanding about the empirical distribution of the rank-order preferences of different items and their popularity. To further understand the data and make inferences about its structure, an efficient method is to establish some statistical models for ranking data. Over the years, various statistical models for ranking data have been developed. In this subsection, we review a commonly used approach, the Luce model.

Suppose *n* judges are asked to rank *k* items. Luce [29] proposed a ranking process where independent utilities *V* = (*V*_*1*_*, V*_*2*_*,..,V*_*k*_) ≥ 0 are assigned to item 1,2, …,*k*. The probability of observing ranking *π*_*n*_ is

$$P\left(\pi |V\right)=\prod_{j=1}^{k-1}\frac{V_{\pi_{n}^{-1}\left(j\right)}}{\sum_{i=j}^{k}V_{\pi_{n}^{-1}\left(i\right)}},$$

and the resulting models is referred to as the Luce models [16]. The Luce models can be interpreted as a vase model [15]: imagine there are infinitely many balls inside a vase, and each ball is labeled *j*., *j* = 1, 2, …, *k*. The proportion of balls labeled with *j* is proportional to *Vj*. Then, the Luce models correspond to the ranking process whereby the first ball drawn is labeled *π*^*-1*^(1), the second ball drawn is labeled *π*^*-1*^(2) (with all balls labeled *π*^*-1*^(1) removed from the vase), and the process continues until all balls in the vase have the same label.

The loglikelihood function is globally concave, and hence a global maximum exists. The MLE of the parameters can thus be obtained using standard methods, e.g., the Newton–Raphson algorithm. Besides MLE, Bayesian method can also be used for parameter estimation, using expectation propagation [30], generalized repeated insertion model [31], and random atomic measures [32].

The Luce model can be extended to incorporate covariates. We can include *M* covaraites of judge *n*, *x*_*mn*_, *m* = 1, 2, …, *M*, into the utilities, that is,

$$V_{\mathrm{nj}}=\beta_{j0}+\sum_{m=1}^{M}\beta_{\mathrm{jm}}x_{\mathrm{nm}},$$

where *β*_*jm*_, *m* = 0, 1, 2, …, *M* are parameters specific to item *j*. This extension of the Luce model is known as the rank-ordered logit (ROL) model [33-35].

### Statistical models for ranking data: distance-based model

In what follows, we will introduce the distance-based model for ranking data. Before doing so, we need to have a clear definition of the “distance” between two rankings. A distance function is useful in measuring the discrepancy between two rankings. The usual properties of a distance function between two rankings *π* and *σ* are:

$$d\left(\pi ,\;\pi \right)=0,$$

$$d\left(\pi ,\;\sigma \right)>0\;\mathrm{if}\;\pi \neq \sigma ,$$

$$d\left(\pi ,\;\sigma \right)=d\left(\sigma ,\;\pi \right).$$

For ranking data, we require that the distance, apart from having these usual properties, must be right invariant, i.e., *d*(*π, σ*) = *d*(*π○γ, σ○γ*), where *π○γ*(*i*) = *π(γ*(*i*)). This requirement ensures that the relabeling of items has no effect on the distance.

Some popular right-invariant distances are Spearman’s rho [36], given by

$$R\left(\pi ,\sigma \right)={\left(\sum_{i=1}^{k}{\left[\pi \left(i\right)-\sigma \left(i\right)\right]}^{2}\right)}^{0.5},$$

Spearman’s rho square, given by

$$R^{2}\left(\pi ,\sigma \right)=\sum_{i=1}^{k}{\left[\pi \left(i\right)-\sigma \left(i\right)\right]}^{2},$$

Spearman’s footrule, given by

$$F\left(\pi ,\sigma \right)=\sum_{i=1}^{k}\left|\pi \left(i\right)-\sigma \left(i\right)\right|,$$

and Kendall’s tau, given by

$$T\left(\pi ,\sigma \right)=\sum_{i<j}I\left\{\left[\pi \left(i\right)-\pi \left(j\right)\right]\left[\sigma \left(i\right)-\sigma \left(j\right)\right]<0\right\},$$

where *I*() is the indicator function. There are other distances applicable to ranking data, and readers can refer to [24] for details.

It is reasonable to assume that there is a modal ranking *π*_0_, and we expect most of the judges to have rankings close to *π*_0_. According to this framework, Diaconis [1] developed a class of distance-based models,

$$P\left(\left(\pi \right|\lambda ,\pi_{0}\right)=\frac{e^{-\mathrm{\lambda d}\left(\pi ,\pi_{0}\right)}}{C\left(\lambda \right)},$$

where *λ* > 0 is the dispersion parameter, *C*(*λ*) is the proportionality constant, and *d*(*π*_,_*π*_0_) is an arbitrary right invariant distance. When we use Kendall’s tau as the distance function, the model is called Mallows’ ϕ-model [37]. In distance-based models, rankings nearer to the modal ranking *π*_0_ have a higher probability of occurrence and this is controlled by *λ*. The distribution of rankings will be more concentrated around *π*_0_ for a smaller value of *λ*.

A closed form for the proportionality constant *C*(*λ*) only exists for some distances. In principle, it can be solved numerically by summing $e^{-\mathrm{\lambda d}\left(\pi ,\;\pi_{0}\right)}$ over all possible *π*. The computational time increases exponentially with the number of items [17].

### Statistical models for ranking data: ϕ-component model

Fligner and Verducci [17] extended the distance-based models by decomposing the distance metric *d*(*π*_,_*σ*) into *k*-1 distance metrics,

(1) $$d\left(\pi ,\sigma \right)=\sum_{i=1}^{k-1}d_{i}\left(\pi ,\sigma \right),$$

where each *d*_*i*_(*π*_,_*σ*)is independent. Both Kendall’s tau and Cayley’s distance [38] can be decomposed in this form, and Fligner and Verducci [17] developed two new classes of ranking models for these, called ϕ-component models and cyclic structure models, respectively.

Fligner and Verducci [17] showed that Kendall’s tau satisfies [1]:

$$T\left(\pi ,\pi_{0}\right)=\sum_{\pi_{0}\left(i\right)=1}^{k-1}V_{\pi_{0}\left(i\right)},$$

where

$$V_{\pi_{0}\left(i\right)}=\sum_{\pi_{0}\left(j\right)=\pi_{0}\left(i\right)+1}^{k}I\left\{\left[\pi \left(i\right)-\pi \left(j\right)\right]>0\right\}.$$

Here, *V*_1_ represents the number of adjacent transpositions required to place the best item in *π*_0_ in the first position. *V*_2_ is the number of adjacent transpositions required to place the second best item in *π*_0_ in the second position, and so on. Therefore, the ranking can be described as *k*-1 stages, *V*_1_ to *V*_*k*-1_, where *V*_*i*_ = *m* can be interpreted as *m* mistakes made in stage *i*.

By applying a dispersion parameter *λ*_*i*_ to stage *V*_*i*_, the Mallows’ ϕ-model is extended to:

$$P\left(\left(\pi \right|\Lambda ,\pi_{0}\right)=\frac{e^{-\sum_{\pi_{0}\left(i\right)=1}^{k-1}\lambda_{\pi_{0}\left(i\right)}V_{\pi_{0}\left(i\right)}}}{C\left(\Lambda \right)},$$

where Λ = {*λ*_*i*_, *i* = 1,…, *k* = 1} and *C*(Λ) is the proportionality constant, which equals

$$\prod_{\pi_{0}\left(i\right)=1}^{k-1}\frac{1-e^{-\left(k-\pi_{0}\left(i\right)+1\right)\lambda_{\pi_{0}\left(i\right)}}}{1-e^{-\lambda_{\pi_{0}\left(i\right)}}}.$$

These models were named *k*-1 parameter models by Fligner and Verducci [17], but were also named ϕ-component models in other papers [24]. Mallows’ ϕ -models are special cases of ϕ-component models when *λ*_1_ = … = *λ*_*k-*1_.

### Statistical models for ranking data: weighted distance-based model

Lee and Yu [18,19] proposed an extension of the distance-based model by replacing the (equal-weighted) distance with a new weighted distance measure, so that different weights can be assigned to different ranks.

Motivated by the weighted Kendall’s tau correlation coefficient [39], Lee and Yu [18,19] defined the weighted Kendall’s tau distance by

$$T_{w}\left(\pi ,\sigma \right)=\sum_{i<j}w_{\pi_{0}\left(i\right)}w_{\pi_{0}\left(j\right)}I\left\{\left[\pi \left(i\right)-\pi \left(j\right)\right]\left[\sigma \left(i\right)-\sigma \left(j\right)\right]<0\right\}.$$

It is important to note that this weighted distance satisfies all the usual distance properties, in particular the symmetry property, i.e., *T*_***w***_(*π*_,_*σ*) = *T*_***w***_(*σ, π*).

Other distance measures can be generalized to a weighted distance in a similar manner to this generalization of Kendall’s tau distance. For example the weighted Spearman’s rho is

$$R_{w}\left(\pi ,\sigma \right)={\left(\sum_{i=1}^{k}w_{\pi_{0}\left(i\right)}{\left[\pi \left(i\right)-\sigma \left(i\right)\right]}^{2}\right)}^{0.5},$$

The weighted Spearman’s rho square is

$$R_{w}^{2}\left(\pi ,\sigma \right)=\sum_{i=1}^{k}w_{\pi_{0}\left(i\right)}{\left[\pi \left(i\right)-\sigma \left(i\right)\right]}^{2},$$

and the weighted Spearman’s footrule is

$$F_{w}\left(\pi ,\sigma \right)=\sum_{i=1}^{k}w_{\pi_{0}\left(i\right)}\left|\pi \left(i\right)-\sigma \left(i\right)\right|.$$

Apart from the weighted Kendall’s tau [39] and weighted Spearman’s rho square [40], many other weighted rank correlations have been proposed [41].

Applying a weighted distance measure *d*_***w***_ to the distance-based model, the probability of observing a ranking *π* becomes

$$P\left(\left(\pi \right|w,\pi_{0}\right)=\frac{e^{-d_{w}\left(\pi ,\pi_{0}\right)}}{C\left(w\right)}.$$

Generally speaking, if *w*_*i*_ is large, few people will tend to disagree that the item ranked *i* in *π*_0_ should not be ranked *i*. This is because such disagreement will greatly increase the distance and hence the probability of observing it will become very small. If *w*_*i*_ is close to zero, people have little or no preference on how the item ranked *i* in *π*_0_ is ranked, because a change in its rank will not affect the distance at all. The extension of weighted distance-based ranking models can retain the nature of distance, and at the same time maintain a greater flexibility. Readers are referred to [19] for the details of these properties.

### Label ranking method using *k*-nearest neighbor algorithm

Label ranking is defined as the problem of classifying a judge’s ranking over a set of items given the covariate of this judge and a training dataset. ROL can be used for this, as it produces utility scores that can generate rankings for the judges. However, when the number of items and covariate are large, ROL may not be feasible due to its long computation time. Recently, a local *k*-nearest neighbor method has been developed for label ranking [42]. If we want to predict the ranking of judge *i*, we can first select the *k*-nearest neighbor (by Euclidean distance) of *i*. Second, a statistical model (the Luce model in [42]) is fitted to these *k* neighbors and the parameters will be used to predict the ranking of judge *i*.

## Results and discussion

In this section, we will use a seven-item ranking dataset *q4*[11], in which 566 Hong Kong physicians ranked the top five incentives (1: competitive pressures; 2: increased savings; 3: government regulation; 4: improved efficiency; 5: improved quality care; 6: patient demand; 7: financial incentives) to the computerization of clinical practice. Items not ranked were imputed using the mean rank. The dataset is not available in the *pmr* package but is available upon request. Note that most of the functions in *pmr* require the input ranking data to be organized in an aggregated format, that is, a summary matrix with rankings and their corresponding frequencies. To transform the individual ranking data to an aggregated format, the *rankagg* function can be used (q4agg < - rankagg(q4)).

All analyses of ranking data start from descriptive statistics. Using the R code destat(q4agg), the *destat* function produces the mean rank vector, the pairs matrix, and the marginal:

From the descriptive statistics, we can deduce that item 4, improved efficiency, is the most preferred item, and item 3, government regulation, is the least preferred item.

Statistical inferences about ranking data can be performed using the *destat* function. For instance, if we want to test whether the ranking over seven items is uniform using mean rank, the following R code can be input:

and would give the output:

The *χ*^2^ test statistic equals 524.8747 and the corresponding *p*-value equals 1.82345 × 10^-110^. Thus, the ranking was not uniformly distributed.

This example illustrates how to test the uniformity of a ranking dataset using the *destat* function, and we will now explain how to compare two ranking datasets using the same function. For example, we may wish to test the hypothesis that physicians with monthly incomes above and below HK$100,000 (rankings stored in *q4agg.highincome* and *q4agg.lowincome* respectively) have different preferences towards computerization incentives. According to the marginal matrix using the *χ*^2^ test, the following codes:

give the output:

The *χ*^2^ test statistic equals 66.415 and the corresponding *p*-value equals 0.04. Thus, we have found a significant difference between physicians’ preferences with respect to their monthly income.

**Figure 2:**
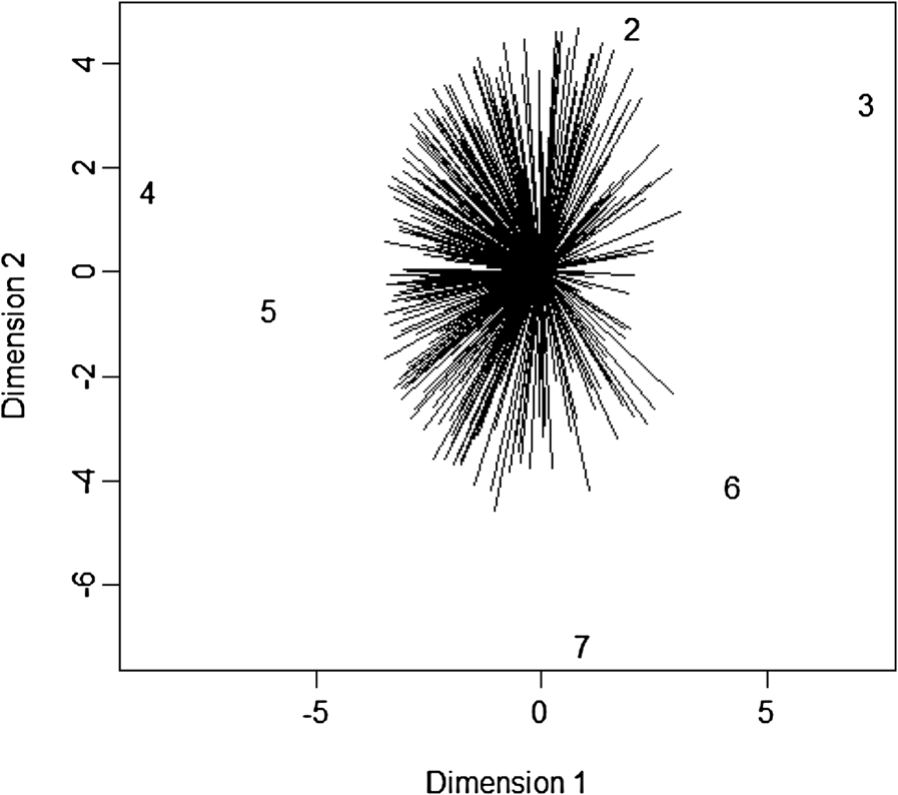
**Multidimensional preference of the *q4 *dataset (1: competitive pressures; 2: increased savings; 3: government regulation; 4: improved efficiency; 5: improved quality care; 6: patient demand; 7: financial incentives).**

Multidimensional preference analysis [28] can help us understand more about the physicians’ ranking process and their preferences over the seven items by decomposing the rankings into a few dimensions. This can be performed using the *mdpref* function (R code: mdpref(q4agg,rank.vector = T)). The output is as follows:

and the 2D plot is given in Figure 2.

The coordinates of the items and rankings, and the proportion of variance explained by the first two dimensions are stored in the values *$item*, *$ranking* and *$explain* respectively. The final two columns of the *$ranking* matrix are the coordinates of the first two columns of $\frac{\mathrm{DV}\prime }{\sqrt{N-1}}$.

Figure 2 shows the multidimensional preference graph. The 2D plot explains around 42 % of the total variance. The first dimension can be interpreted as the overall preference of the seven items (labeled as “internal/external”). The leftmost item (item 4) and rightmost item (item 3) are the most and the least preferred items, respectively. The second dimension can be interpreted as the overall variance of the seven items (labeled as “push/pull factors”). The bottommost item (item 7) has the largest variance and the topmost item (item 2) has the second largest variance among the seven items.

Descriptive statistics and plots provide an insight to the data, but modeling will be more useful if we wish to have a deeper understanding. The Luce model (*pl*), distance-based model (*dbm*), ϕ-component model (*phicom*) and weighted distance-based model (*wdbm*) can be fitted using the *pmr*, which requires the *stats4* package. We will demonstrate the model fitting procedure. Spearman’s footrule distance usually gives the best fit [18,19] and hence it will be used in our demonstration of distance-based models.

The parameter estimates of the Luce model can be obtained using the R code q4.pr <- pl(q4agg); q4.pr@coef, and the output is as follows:

The warning messages are a result of some of the predicted probabilities being close to zero. The parameter estimates of the distance-based model can be obtained using the R code q4.dbm < - dbm(q4agg); q4.dbm@coef, and the distance type can be specified using the argument *dtype* (default: Kendall’s tau; *rho*: Spearman’s rho; *rho2*: Spearman’s rho square; *foot*: Spearman’s footrule).

The loglikelihood is a suitable criterion for determining which model should be used. The model with the largest loglikelihood is selected. We can compute the loglikelihood of all models using the minimum value (*@min*) of the negative loglikelihood function, which is built-in for maximum likelihood models:

and the output is as follows:

The best model (with the smallest negative loglikelihood) is the weighted footrule model. The parameters are given by the R code q4.wdbm@coef as follows:

From the model parameters, we can conclude that item 4 is ranked 1^*st*^, but the judges preference for this position is not particularly strong. Note that the modal ranking in the weighted distance-based model is different from that using the mean rank.

As the “best” model does not imply that it gives an adequate fit to the data, we need to assess the goodness-of-fit. The sum of squares Pearson residuals (*χ*^2^) [18,19] can be used for this purpose, and is provided in *pmr*. It is given by

$$\chi^{2}=\sum_{i}^{k!}r_{i}^{2},$$

where $r_{i}=\frac{O_{i}-E_{i}}{\sqrt{E_{i}}}$ is the Pearson residual, and *O*_*i*_, *E*_*i*_ are the observed and expected frequencies of ranking *i*, respectively. The sum of square Pearson residual will automatically be given in the output, together with the corresponding degrees of freedom.

We can also examine the effect of physicians’ gender and type (private/public) on their preferences (gender and type stored in *q4cov*) using the ROL model. This can be fitted using the *rol* function in the *pmr* package with the R code q4.rol <- rol(q4,q4cov); q4.rol@coef where *covariate* stores the gender and type of every physicians. The output is as follows:

These parameters are difficult to interpret without their corresponding significance levels. To obtain the *p*-values, the following R code can be used:

which gives the output:

According to the results of the ROL model, female physicians preferred items 1 and 4, and private physicians did not prefer items 1, 2, and 7.

Assume that we want to predict the preference of a list of physicians with known covariates *q4covtest*. One possible method is to assign the utility ranks of the seven items for these physicians using the parameters obtained from the ROL model. Another method is to use the local *k*-nearest neighbor algorithm with the R code local.knn(q4,q4covtest,q4cov,knn.k = k). The value of *k* must be pre-specified. The *pmr* package provides the cross-validation version of the local k-nearest neighbor local.knn.cv(q4,q4covtest,q4cov). By default this uses 10-fold cross validation and tests the cross-validation prediction error of *k* (defined as the total Kendall’s distance) from 1 to 20.

## Conclusions

In this paper, we presented the *pmr* R package, the first package for analyzing and modeling ranking data. The package provides insight to users through descriptive statistics of ranking data. Users can also visualize ranking data by applying a thought multidimensional preference analysis. Various probability models for ranking data are also included, allowing users to choose that which is most suitable to their specific situations. Besides the models introduced in this paper, there are other functions included in the *pmr* package that have not been presented here due to scope limitations, including the Analytic Hierarchy Process model (*ahp*) [26,43], multidimensional preference analysis (*mdpref*), and rank plots (*rankplot*) [44]. Details of these functions can be found at http://cran.r-project.org/web/packages/pmr/pmr.pdf. Future works on developing the package will include the incorporation of latent class models.

In the *pmr* package, we aimed at including traditional ranking models like the Luce model and distance-based model, and many recently-developed models for ranking data were not included (examples included decision tree models for ranking data [18,45,46] and multistage models [47,48]). Nevertheless, since many of these models belong to extensions of traditional ranking models, we believe that the development of new ranking models can rely on the programming code provided by package *pmr*.

## Availability and requirements

Project name: Probability Models for Ranking Data

Project home page: http://cran.r-project.org/web/packages/pmr/index.html

Operating system(s): Platform independent

Programming language: R

Other requirements: R 2.15.0 or above

License: GPL-2

Any restrictions to use by non-academics: none

## Competing interests

The authors declare that they have no competing of interests.

## Authors’ contributions

PHL wrote the package *pmr* and drafted the manuscript. PLHY helped in the development of the package *pmr* and significantly revised the manuscript. All authors read and approved the final manuscript.

## Pre-publication history

The pre-publication history for this paper can be accessed here:

http://www.biomedcentral.com/1471-2288/13/65/prepub

## Supplementary Material

Additional file 1 Package source of package pmr. Click here for file

Additional file 2 Reference manual of package pmr. Click here for file
