# Supplementary material for: An R package for analyzing and modeling ranking data
Source: BMC Med Res Methodol. 2013 May 14;13:65. doi: 10.1186/1471-2288-13-65 (PMC3665468; doi:10.1186/1471-2288-13-65)
Supplement: Additional file 2 — Reference manual of package pmr. [file 1471-2288-13-65-S2.pdf]

Package ‘pmr’

February 1, 2013

Type Package

Title Probability Models for Ranking Data

Version 1.2.0

Date 2013-01-16

Depends stats4,graphics

Author Paul H. Lee and Philip L. H. Yu

Maintainer Paul H. Lee <honglee@graduate.hku.hk>

Description Descriptive statistics (mean rank, pairwise frequencies, and marginal matrix), Analytic Hierarchy Process models (with Saaty’s and Koczkodaj’s inconsistencies), probability models (Luce models, distance-based models, and rank-ordered logt models) and visualization with multimendional preference analysis for ranking data are provided. Current, only complete rankings are supported by this package.

License GPL-2

LazyLoad yes

R topics documented:

ahp 2
big4 3
breasttissue 4
dbm 5
destat 6
idea 6
leisure.black 7
leisure.white 8
local.knn 9
local.knn.cv 10
mdpref 11
phicom 12
pl 13
Probability Models for Ranking Data 14
rankagg 14
rankplot 15

rinv 16
rol 17
song 18
wdbm 19

Index **20**

---

ahp *The Analytic hierarchy process (AHP)*

Description

Computing the weights, Saaty's (1977) and Koczkodaj's (1997) inconsistencies for analytic hierarchy process (AHP).

Usage

```
ahp(dset, sim_size=500)
```

Arguments

`dset`	an "A" matrix. It should be a square matrix with diagonal values equal 1 and \$a_ij = 1/a_ij\$.
`sim_size`	simulation size for computation of Saaty's inconsistency. Default is 500.

Value

`weighting`	Eigenvalues of the criteria.
`Saaty`	Saaty's inconsistency.
`Koczkodaj`	Koczkodaj's inconsistency.

Author(s)

Paul H. Lee and Philip L. H. Yu

References

Koczkodaj, W. W., Herman, M. W., and Orlowski, M. (1997) Using consistency-driven pairwise comaprisons in knowledge-based systems. Proceedings of the sixth international conference on information and knowledge management, ACM Press, 91-96.

Saaty, T. L. (1980) The Analytic Hierarchy Process. McGraw-Hill, New York.

Saaty, T. L. (1977) A scaling methods for priorities in hierarchical structure. Journal of Mathematical Psychology, 15, 234-281.

Examples

```
## create an artificial A matrix abc, example taken from Koczkodaj et al. (1997)
abc <- matrix(data = 1:15, nrow = 4, ncol = 4, byrow = TRUE)
abc[1,1] <- 1
abc[1,2] <- 2
abc[1,3] <- 5
abc[1,4] <- 4
abc[2,1] <- 1/2
abc[2,2] <- 1
abc[2,3] <- 3
abc[2,4] <- 1.9
abc[3,1] <- 1/5
abc[3,2] <- 1/3
abc[3,3] <- 1
abc[3,4] <- 0.7
abc[4,1] <- 1/4
abc[4,2] <- 1/1.9
abc[4,3] <- 1/0.7
abc[4,4] <- 1

## compute the weights, Saaty's and Koczkodaj's inconsistencies
## ahp(abc)
```

---

big4 *big4*

Description

Ranking of 4 English Premier League teams from season 1992-1993 to 2009-2010.

Usage

```
data(big4)
```

Format

A data frame with 18 observations on the following 5 variables.

`Arsenal` the rank of team "Arsenal"

`Chelsea` the rank of team "Chelsea"

`Liverpool` the rank of team "Liverpool"

`Manchester.United` the rank of team "Manchester United"

`n` number of observations

Details

The comparative performance of the "Big Four" since the start of the English Premier League.

Source

Wikipedia. (2010) http://en.wikipedia.org/wiki/Premier_League

References

Wikipedia. (2010) http://en.wikipedia.org/wiki/Premier_League

Examples

```
data(big4)
## maybe str(big4) ; plot(big4) ...
```

`breasttissue` *breasttissue*

Description

Ranking of 4 breast cancer categories of 106 observations. The original dataset was a categorical one, and it was converted to the current ranking dataset by sorting the expected probabilities (computed by fitting a stepwise logistic regression) of the 4 categories.

Usage

```
data(breasttissue)
```

Format

A data frame with 106 observations on the following 4 variables.

`adi` the rank of category "adipose"

`car` the rank of category "carcinoma"

`con` the rank of category "connective"

`new` the rank of cateogry "fibro-adenoma, mastopathy or glandular"

Details

The rankings were generated by first fitting a stepwise logistic regression to the original dataset, and then sorted by the expected probabilities of the 4 categories.

Source

Frank, A., Asuncion, A. (2010) UCI Machine Learning Repository. http://archive.ics.uci.edu/ml

References

Jossinet, J. (1996) Variability of impedivity in normal and pathological breast tissue. Medical and Biological Engineering and Computing 34, 346-350.

da Silva, J. E., de Sa, J. P., Jossinet, J. (2000) Classification of breast tissue by electrical impedance spectroscopy. Medical and Biological Engineering and Computing 38, 26-30.

Examples

```
data(breasttissue)
## maybe str(breasttissue) ; rankagg(breasttissue) ...
```

`dbm` *Distance-based Models*

Description

Distance-based Models for ranking data. The distance-based models assume that rankings closer to the modal ranking are more likely to be observed.

Usage

```
dbm(dset, dtype="tau")
```

Arguments

`dset`	a ranking dataset
`dtype`	type of distance between two rankings. tau : Kendall's tau, rho : Spearman's rho, rho2 : Spearman's rho square, foot : footrule

Details

Fit the distance-based models for the dataset and return a mle object. Standard methods on mle (e.g., @coef, @vcov) apply. The modal ranking and the Chi-square residual are given in the output.

Author(s)

Paul H. Lee and Philip L. H. Yu

See Also

`wdbm`

Examples

```
library(pmr)
## create an artificial dataset
X1 <- c(1,1,2,2,3,3)
X2 <- c(2,3,1,3,1,2)
X3 <- c(3,2,3,1,2,1)
n <- c(6,5,4,3,2,1)
test <- data.frame(X1,X2,X3,n)

## fit the distance-based model with Spearman's rho distance
## dbm(test,dtype="rho")
```

destat — *Descriptive statistics of a ranking dataset*

Description

Computing the descriptive statistics (mean rank, pairs matrix, and marginals matrix) of a ranking dataset.

Usage

```
destat(dset)
```

Arguments

dset	a ranking dataset (aggregated)

Value

mean.rank	Mean rank of the items.
pair	The number of observations which the first item (row) is more preferred than the second item (column).
mar	The number of observations which the item i (row) is ranked j (column).

Author(s)

Paul H. Lee and Philip L. H. Yu

Examples

```
## create an artificial dataset
X1 <- c(1,1,2,2,3,3)
X2 <- c(2,3,1,3,1,2)
X3 <- c(3,2,3,1,2,1)
n <- c(6,5,4,3,2,1)
test <- data.frame(X1,X2,X3,n)

## compute the descriptive statistics of the artificial dataset
## destat(test)
```

idea — *idea*

Description

Ranking of 5 items according to the similarity with the word "idea".

Usage

```
data(idea)
```

Format

A data frame with 98 observations on the following 6 variables.

X1 the rank of word "thought"

X2 the rank of word "play"

X3 the rank of word "theory"

X4 the rank of word "dream"

X5 the rank of word "attention"

n number of observations

Details

98 college students were asked to rank five words according to the similarity with the word "idea". The five words were (1) thought, (2) play, (3) theory, (4) dream, and (5) attention.

Source

Fligner, M. A., and Verducci, J. S. (1986) Distance based ranking models. Journal of the Royal Statistical Society Series B, 48(3), 359-369.

References

Fligner, M. A., and Verducci, J. S. (1986) Distance based ranking models. Journal of the Royal Statistical Society Series B, 48(3), 359-369.

Examples

```
data(idea)
## maybe str(idea) ; plot(idea) ...
```

`leisure.black` *leisure.black*

Description

Ranking of 3 items according to the preference of the group in an activity.

Usage

```
data(leisure.black)
```

Format

A data frame with 13 observations on the following 4 variables.

X1 Male

X2 Female

X3 Both

n number of observations

Details

13 black women, aged 70-79, were asked whom they would prefer to spend their leisure time.

Source

Hollander, M. and Sethuraman, J. (1978) Testing for agreement between groups of judges. Biometrika, 65, 403-411.

References

Marden, J. I. (1995) Analyzing and Modeling Rank Data. Chapman and Hall.

Examples

```
data(leisure.black)
## maybe str(leisure.black) ; plot(leisure.black) ...
```

---

leisure.white *leisure.white*

Description

Ranking of 3 items according to the preference of the group in an activity.

Usage

```
data(leisure.white)
```

Format

A data frame with 14 observations on the following 4 variables.

`X1` Male

`X2` Female

`X3` Both

`n` number of observations

Details

14 white women, aged 70-79, were asked whom they would prefer to spend their leisure time.

Source

Hollander, M. and Sethuraman, J. (1978) Testing for agreement between groups of judges. Biometrika, 65, 403-411.

References

Marden, J. I. (1995) Analyzing and Modeling Rank Data. Chapman and Hall.

Examples

```
data(leisure.white)
## maybe str(leisure.white) ; plot(leisure.white) ...
```

`local.knn` *Local k-nearest neighbor method for label ranking.*

Description

Predict the ranking of a group of judges based on a training dataset with rankings and covariates. First, for each judge, the k-nearest neighbors (by Euclidean distance) are selected. Second, the prediction of rankings are done based on the rankings of these neighbors. Users can chooce two methods of prediction: by mean rank or by Luce model.

Usage

```
local.knn(dset,covariate.test,covariate,knn.k=1,method="mean")
```

Arguments

`dset`	a ranking dataset for training the k-nearest neighbor.
`covariate.test`	the covariates of the judges to be predicted.
`covariate`	the covariates of the rankings.
`knn.k`	the number of nearest neighbors to be included. The default value is 1.
`method`	the prediction method. mean : mean rank, pl : Luce model

Author(s)

Paul H. Lee and Philip L. H. Yu

References

Cheng, W., Dembczynski, K., Hullermeier, E. (2010). Label ranking methods based on the Plackett-Luce model. Proceedings of ICML 2010.

See Also

`local.knn.cv`

Examples

```
## create an artificial dataset
X1 <- c(1,1,2,2,3,3)
X2 <- c(2,3,1,3,1,2)
X3 <- c(3,2,3,1,2,1)
co <- c(6,5,4,3,2,1)
co.test <- 1.2
train <- data.frame(X1,X2,X3)

## local k-nearest neighbor method of the artificial dataset
## local.knn(train,co.test,co)
```

`local.knn.cv` *Local k-nearest neighbor method for label ranking.*

Description

Local k-nearest neighbor method with the parameter k determined using cross-validation error (defined as the sum of Kendall's distance).

Usage

```
local.knn.cv(dset,covariate.test,covariate,cv=10,k.max=20,method.cv="mean")
```

Arguments

`dset`	a ranking dataset for training the k-nearest neighbor.
`covariate.test`	the covariates of the judges to be predicted.
`covariate`	the covariates of the rankings.
`cv`	the number of cross-validated samples. The default value is 10.
`k.max`	the maximum number of nearest neighbors to be tested. The default value is 20.
`method.cv`	the prediction method. mean : mean rank, pl : Luce model

Author(s)

Paul H. Lee and Philip L. H. Yu

References

Cheng, W., Dembczynski, K., Hullermeier, E. (2010). Label ranking methods based on the Plackett-Luce model. Proceedings of ICML 2010.

See Also

`local.knn`

Examples

```
## create an artificial dataset
X1 <- c(1,1,2,2,3,3)
X2 <- c(2,3,1,3,1,2)
X3 <- c(3,2,3,1,2,1)
co <- c(6,5,4,3,2,1)
co.test <- 1.2
train <- data.frame(X1,X2,X3)

## local k-nearest neighbor method of the artificial dataset
## local.knn.cv(train,co.test,co)
```

mdpref *Multidimensional preference analysis.*

Description

Display a 2D plot of the position of both judges and items. The items are labeled with consecutive numbers 1, 2, ..., k while the judges are presented as vectors pointing from the origin to their most preferred items.

Usage

```
mdpref(dset,rank.vector=FALSE)
```

Arguments

`dset`	a ranking dataset
`rank.vector`	The vectors of the rankings at default will be displayed if the value is set to TRUE.

Details

Multidimenional preference analysis is a dimension reduction technique which aims to project the high-dimensional ranking data into 2D or 3D plot. Dimension reduction is done using singular value decomposition. Note that the perpendicular projection of the item points onto a judge vector represents the ranking of these items by this judge.

Value

`item`	Coordinates of the items.
`ranking`	Coordinates of the rankings.
`explain`	Proportion of variance explained by the first two dimensions.

Author(s)

Paul H. Lee and Philip L. H. Yu

References

Carroll, J. D. (1972) Individual differences and multidimensional scaling. In Shepard, R. N., Romney, A. K., and Nerlove, S. B. (eds.)

Examples

```
## create an artificial dataset
X1 <- c(1,1,2,2,3,3)
X2 <- c(2,3,1,3,1,2)
X3 <- c(3,2,3,1,2,1)
n <- c(6,5,4,3,2,1)
test <- data.frame(X1,X2,X3,n)

## multidimensional preference analysis of the artificial dataset
## mdpref(test,rank.vector=TRUE)
```

phicom *phi-component Models*

Description

phi-component Models for ranking data. The distance-based models assume that rankings closer to the modal ranking are more likely to be observed. Phi-component models are extensions of distance-based models with Kendall's distance by allowing weights at different stages.

Usage

```
phicom(dset)
```

Arguments

dset	a ranking dataset (aggregated)

Details

Fit the phi-component models for the dataset and return a mle object. Standard methods on mle (e.g., @coef, @vcov) apply. The modal ranking and the Chi-square residual are given in the output.

Author(s)

Paul H. Lee and Philip L. H. Yu

References

Fligner, M. A., and Verducci, J. S. (1986) Distance based ranking models. Journal of the Royal Statistical Society Series B, 48(3), 359-369.

See Also

dbm

Examples

```
## create an artificial dataset
X1 <- c(1,1,2,2,3,3)
X2 <- c(2,3,1,3,1,2)
X3 <- c(3,2,3,1,2,1)
n <- c(6,5,4,3,2,1)
test <- data.frame(X1,X2,X3,n)

## fit the phi-component model
## phicom(test)
```

pl *The Luce Models*

Description

The Luce Models for ranking data. The Luce models assumed that the rankings of the items are proportional to the item parameters.

Usage

```
pl(dset)
```

Arguments

dset	a ranking dataset (aggregated)

Details

Fit the Luce models for the dataset and return a mle object. Standard methods on mle (e.g., @coef, @vcov) apply. The Chi-square residual are given in the output.

Author(s)

Paul H. Lee and Philip L. H. Yu

References

Luce, R. D. (1959) Individual Choice Behavior. New York: Wiley.

See Also

rinv

Examples

```
## create an artificial dataset
X1 <- c(1,1,2,2,3,3)
X2 <- c(2,3,1,3,1,2)
X3 <- c(3,2,3,1,2,1)
n <- c(6,5,4,3,2,1)
test <- data.frame(X1,X2,X3,n)

## fit the Luce model
## pl(test)
```

`Probability Models for Ranking Data` *Probability Models for Ranking Data*

Description

Probability models for ranking data.

Details

Package:	pmr
Type:	Package
Version:	1.2.0
Date:	2013-01-16
Depends:	stats4, graphics
License:	GPL-2
LazyLoad:	yes

This package includes various probability models for ranking data. Current, only complete rankings are supported by this package.

Author(s)

Paul H. Lee and Philip L. H. Yu

Maintainer: Paul H. Lee <honglee@graduate.hku.hk>

References

Marden, J. I. (1995) Analyzing and Modeling Rank Data. Chapman and Hall.

`rankagg` *Summary of a ranking dataset.*

Description

Turn individual rankings into a summary matrix.

Usage

```
rankagg(dset)
```

Arguments

`dset` each row represent a single ranking

Value

Return the dataset which summarize the original ranking data.

Author(s)

Paul H. Lee and Philip L. H. Yu

Examples

```
## create an artificial dataset
X1 <- c(1,1,2,2,3,3)
X2 <- c(2,3,1,3,1,2)
X3 <- c(3,2,3,1,2,1)
test <- data.frame(X1,X2,X3)

## aggregate the ranking of all the observations and create a summary matrix
## test2 <- rankagg(test)
```

---

rankplot *Visualizing rankings of 3 or 4 items by plot.*

Description

Display rankings of 3 items by hexagon and 4 items by truncated octahedron. Each line linking two points represents a Kendall distance of one. The size of the point is proportional to the frequency of that particular ranking.

Usage

```
rankplot(dset,trans=FALSE)
```

Arguments

`dset`	a ranking dataset
`trans`	(For 4-item ranking dataset) Another representation of the rankings.

Details

As a ranking dataset of k items can be represented in k-1 dimensions, ranking data with 3 or 4 items can be represented in graphs without losing any information. For visualization of ranking data with more items, multidimensional preference analysis can be used.

Author(s)

Paul H. Lee and Philip L. H. Yu

References

Thompson, G. L. (1993). Graphical techniques for ranked data. In Fligner, M. A., and Verducci, J. S. (eds.) Probability Models and Statistical Analyses for Ranking Data, pp. 294-298.

Examples

```
## create an artificial dataset
X1 <- c(1,1,2,2,3,3)
X2 <- c(2,3,1,3,1,2)
X3 <- c(3,2,3,1,2,1)
n <- c(6,5,4,3,2,1)
test <- data.frame(X1,X2,X3,n)

## multidimensional preference analysis of the artificial dataset
## rankplot(test)
```

`rinv` *Inverse of a ranking dataset.*

Description

Computing the inverse of a ranking dataset.

Usage

```
rinv(dset)
```

Arguments

`dset`	a ranking dataset

Value

Return the dataset which is the orderings of the original ranking data.

Author(s)

Paul H. Lee and Philip L. H. Yu

Examples

```
## create an artificial dataset
X1 <- c(1,1,2,2,3,3)
X2 <- c(2,3,1,3,1,2)
X3 <- c(3,2,3,1,2,1)
n <- c(6,5,4,3,2,1)
test <- data.frame(X1,X2,X3,n)

## compute the inverse of the artificial dataset
## rinv(test)
```

rol *The Rank-ordered Logit Models*

Description

The Rank-ordered Logit (ROL) Models for ranking data. ROL models are extensions of the Luce models by incorporating covariates.

Usage

```
rol(dset, covariate)
```

Arguments

dset	a ranking dataset
covariate	the covariates of the ranking dataset

Details

Fit the rank-ordered logit models for the dataset and return a mle object. Standard methods on mle (e.g., @coef, @vcov) apply. By default, the intercept term is included.

Author(s)

Paul H. Lee and Philip L. H. Yu

References

Beggs, S., Cardell, S., and Hausman, J. (1981) Assessing the potential demand for electric cars. Journal of Econometrics, 16: 1-19.

Chapman, R. G., and Staelin, R. (1982) Exploiting rank ordered choice set data within the stochastic utility model. Journal of Market Research, 19:288-301.

Hausman, J., and Ruud, P. A. (1987) Specifying and testing econometric models for rank-ordered data. Journal of Econometrics, 34:83-104.

See Also

pl

Examples

```
## create an artificial dataset
X1 <- c(1,1,2,2,3,3)
X2 <- c(2,3,1,3,1,2)
X3 <- c(3,2,3,1,2,1)
X4 <- c(6,5,4,3,2,1)
test <- data.frame(X1,X2,X3)

## fit the Luce model
## rol(test,X4)
```

song *song*

Description

Ranking of 5 items according to the similarity with the word "song".

Usage

```
data(song)
```

Format

A data frame with 98 observations on the following 6 variables.

- `X1` the rank of word "score"
- `X2` the rank of word "instrument"
- `X3` the rank of word "solo"
- `X4` the rank of word "benediction"
- `X5` the rank of word "suit"
- `n` number of observations

Details

83 college students were asked to rank five words according to the similarity with the word "song". The five words were (1) score, (2) instrument, (3) solo, (4) benediction, and (5) suit.

Source

Critchlow, D. E., Fligner, M. A., and Verducci, J. S. (1991) Probability models on rakning. Journal of Mathematical Psychology, 35, 294-318.

References

Critchlow, D. E., Fligner, M. A., and Verducci, J. S. (1991) Probability models on rakning. Journal of Mathematical Psychology, 35, 294-318.

Examples

```
data(song)
## maybe str(song) ; plot(song) ...
```

wdbm *Weighted Distance-based Models*

Description

Weighted Distance-based Models for ranking data. The distance-based models assume that rankings closer to the modal ranking are more likely to be observed. Weighted distance-based models are extensions of distance-based models with by allowing weights for different items.

Usage

```
wdbm(dset, dtype="tau")
```

Arguments

dset	a ranking dataset (aggregated)
dtype	type of weighted distance between two rankings. tau : Kendall's tau, rho : Spearman's rho, rho2 : Spearman's rho square, foot : footrule

Details

Fit the weighted distance-based models for the dataset and return a mle object. Standard methods on mle (e.g., @coef, @vcov) apply. The modal ranking and the Chi-square residual are given in the output.

Author(s)

Paul H. Lee and Philip L. H. Yu

References

Lee, P. H., and Yu, P. L. H. (2010) Distance-based tree models for ranking data. Computational Statistics and Data Analysis, 54(6), 1672-1682.

Lee, P. H., and Yu, P. L. H. (2012) Mixtures of weighted distance-based models for ranking data with applications in political studies. Computational Statistics and Data Analysis, 56(8), 2486-2500.

See Also

dbm

Examples

```
## create an artificial dataset
X1 <- c(1,1,2,2,3,3)
X2 <- c(2,3,1,3,1,2)
X3 <- c(3,2,3,1,2,1)
n <- c(6,5,4,3,2,1)
test <- data.frame(X1,X2,X3,n)

## fit the weighted distance-based model with Spearman's foot distance
## wdbm(test,dtype="foot")
```

Index

∗Topic **Analytic hierarchy process**
  ahp, 2
∗Topic **Luce models**
  pl, 13
∗Topic **Rank-order logit models**
  rol, 17
∗Topic **datasets**
  big4, 3
  breasttissue, 4
  idea, 6
  leisure.black, 7
  leisure.white, 8
  song, 18
∗Topic **destat**
  destat, 6
∗Topic **distance-based model**
  dbm, 5
∗Topic **local.knn.cv**
  local.knn.cv, 10
∗Topic **local.knn**
  local.knn, 9
∗Topic **mdpref**
  mdpref, 11
∗Topic **phi-component model**
  phicom, 12
∗Topic **rankagg**
  rankagg, 14
∗Topic **ranking data**
  Probability Models for Ranking Data, 14
∗Topic **rankplot**
  rankplot, 15
∗Topic **rinv**
  rinv, 16
∗Topic **weighted distance-base models**
  wdbm, 19

ahp, 2

big4, 3
breasttissue, 4

dbm, 5, *12*, *19*
destat, 6

idea, 6

leisure.black, 7
leisure.white, 8
local.knn, 9, *10*
local.knn.cv, *9*, 10

mdpref, 11

phicom, 12
pl, 13, *17*
pmr *(*Probability Models for Ranking Data*)*, 14
pmr-package *(*Probability Models for Ranking Data*)*, 14
Probability Models for Ranking Data, 14

rankagg, 14
rankplot, 15
rinv, *13*, 16
rol, 17

song, 18

wdbm, *5*, 19
